# A Component-Based Study of the Effect of Diameter on Bond and Anchorage Characteristics of Blind-Bolted Connections

**DOI:** 10.1371/journal.pone.0149490

**Published:** 2016-02-22

**Authors:** Muhammad Nasir Amin, Salman Zaheer, Abdulrahman Ali Alazba, Muhammad Umair Saleem, Muhammad Umar Khan Niazi, Nauman Khurram, Muhammad Tahir Amin

**Affiliations:** 1 Department of Civil and Environmental Engineering, College of Engineering, King Faisal University (KFU), P. O. Box 380, Al-Hofuf, Al-Ahsa 31982, Kingdom of Saudi Arabia; 2 Department of Civil Engineering, University of Nottingham, Nottingham, United Kingdom; 3 Alamoudi Water Research Chair, King Saud University, P.O. Box 2460, Riyadh 11451, Kingdom of Saudi Arabia; 4 Department of Environmental Sciences, COMSATS Institute of Information Technology, Abbottabad, 22060, Pakistan; Seoul National University, REPUBLIC OF KOREA

## Abstract

Structural hollow sections are gaining worldwide importance due to their structural and architectural advantages over open steel sections. The only obstacle to their use is their connection with other structural members. To overcome the obstacle of tightening the bolt from one side has given birth to the concept of blind bolts. Blind bolts, being the practical solution to the connection hindrance for the use of hollow and concrete filled hollow sections play a vital role. Flowdrill, the Huck High Strength Blind Bolt and the Lindapter Hollobolt are the well-known commercially available blind bolts. Although the development of blind bolts has largely resolved this issue, the use of structural hollow sections remains limited to shear resistance. Therefore, a new modified version of the blind bolt, known as the “Extended Hollo-Bolt” (EHB) due to its enhanced capacity for bonding with concrete, can overcome the issue of low moment resistance capacity associated with blind-bolted connections. The load transfer mechanism of this recently developed blind bolt remains unclear, however. This study uses a parametric approach to characterising the EHB, using diameter as the variable parameter. Stiffness and load-carrying capacity were evaluated at two different bolt sizes. To investigate the load transfer mechanism, a component-based study of the bond and anchorage characteristics was performed by breaking down the EHB into its components. The results of the study provide insight into the load transfer mechanism of the blind bolt in question. The proposed component-based model was validated by a spring model, through which the stiffness of the EHB was compared to that of its components combined. The combined stiffness of the components was found to be roughly equivalent to that of the EHB as a whole, validating the use of this component-based approach.

## Introduction

Steel is increasingly used around the globe for construction purposes, and is replacing concrete as the main structural material owing to several advantages, including rapid construction and the wide variety of attainable structural shapes. Normally open sections (such as I-shaped, T-shaped or angle irons) were commonly used until a few years ago. More recently, steel structures with hollow section elements have been an attractive choice for several design solutions due to their structural and architectural potential [1]. The advantages of structural hollow sections (SHS) include a high strength-to-weight ratio; greater axial load-carrying capacity; the ability to be used as a composite structural member in the form of steel hollow sections filled with concrete, which further increases the strength and load-carrying capacity of the structural element; reduced vulnerability to corrosion (as no naked edge is present); and ease of paint application. Furthermore, concrete-filled steel tubular (CFST) columns confer excellent structural and constructional benefits, including enhanced stiffness and ductility, and good fire resistance [2]. The steel tube provides confinement, and thus increases the stiffness and strength of the concrete, eliminating the need for formwork during construction [3].

The only constraint to the use of SHS is their connections with other members. The conventional standard bolting arrangement cannot be used in their case as there is no access to the other side of the bolt for tightening [3]. Solutions such as provision of plates or brackets may be efficient but would diminish the aesthetic qualities of hollow steel members. Provision of welds is also an option, but similarly affects aesthetics, workability and site operation. Research into overcoming the obstacle of tightening the bolt from one side has given rise to the concept of blind bolts. The advent of blind-bolted connections has made it possible for designers to generate unique and complex structural designs with improved aesthetics and structural integrity.

Blind bolts, which represent a practical solution to the connection problems associated with hollow and concrete-filled hollow sections, play a vital role. Currently, the use of blind-bolt systems is restricted to shear-resisting joints only. A modified form of the Hollo-Bolt, known as the Reverse Mechanism Hollo-Bolt (RMH), showed a significant improvement in the clamping force between the connected plies, compared with the original arrangement, in a series of different tests [4]. However, sudden failure of the expanding sleeves has limited the commercial use of the RMH. Further attempts to increase the moment-resisting capacity of the blind-bolted joints gave rise to the idea of the Extended Hollo-Bolt (EHB), which has greatly enhanced the moment-resisting capacity within the blind bolt family, and has solved the issue of poor structural integrity. The bolt was designed explicitly for concrete-filled hollow sections [5–7]. Due to the increased length of the bolt shank and the provision of an anchorage mechanism, the blind bolt now has enhanced moment-resisting capacity when used with concrete-filled hollow sections. The EHB confers a stronger bond and improved anchorage in the case of concrete-filled hollow steel sections, and various modifications can be made, depending on the structural requirements [8].

However, the underlying mechanism of this newly developed bolt have not been fully elucidated, and the performance of its components should be studied in detail to achieve a moment resisting blind bolted connection. The characteristics of the EHB can be understood by studying the effect of load on its individual components and their behaviour; hence, further enhancements can be incorporated into its design. The concept of blind bolts is still relatively new, and it is essential to conduct extensive research into all of its parameters to fully understand its load transfer mechanism and behaviour under all practical conditions. Studying the behaviour of its individual components deepens the understanding of the actual mechanism of load transfer in the newly developed blind bolt, facilitating the development of models for its design.

This study focuses on the case of an EHB embedded in concrete-filled SHS. An EHB resists load predominantly through the expanding sleeves, bolt shank and anchor head. This research consists of a component-based in-depth study of the EHB, and a comparison of the EHB as a whole with its individual load-carrying components. It additionally puts forward a parametric approach to characterising the EHB using bolt diameter as the variable parameter.

As mentioned above, the three components (expanding sleeves, bolt shank and anchor head) of EHB, when combined appropriately, should produce an overall effect identical to the EHB itself. In the case of the Hollo-Bolt, the bolt length is too short to achieve any significant bonding with the surrounding concrete, and load is predominantly carried by the expanding sleeves. Thus, the Hollo-Bolt itself can be considered as the expanding sleeves component of the EHB. In the case of the standard bolt shank, where the anchor head is attached at the bottom, the load is predominantly carried by the bond between the bolt and the concrete, and the anchorage is provided by the anchor head. Consequently, the bolt shank and anchor head mechanism can be considered as the bond and anchorage component of the EHB.

## Materials and Methods

For the component-based study of the EHB, force-displacement curves were obtained via pull-out testing of both the EHB as a whole and its individual components (i.e., the Hollo-Bolt, and the bond and anchorage component). The results obtained from the testing of the components were then combined and compared to that of EHB.

For parametric analysis of the EHB, two bolt sizes were chosen. Separate pull-out tests for the material properties of the bolt were performed to determine its modulus of elasticity, yield and fracture points. Also, another test for determining the preload on the bolt was carried out to measure the induced preload in the bolts.

### Bolt sizes and properties

**Fig 1:**
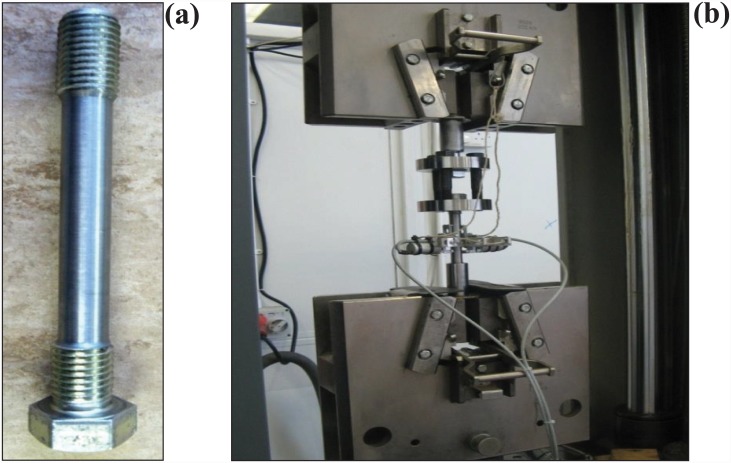
(a) Machined bolt for the tensile test, (b) Test setup for tensile test for M-20 bolt.

The bolt sizes chosen for this study were the M-16 and M-20. The M-20 bolts were tested in-house, while data for the M-16 bolts were obtained from a previously published study [9]. The grade of both bolts was kept constant at 8.8. A tensile test was conducted on a standard M-20 bolt to determine the bolt parameters. The threads of the M-20 standard bolt were removed mechanically to allow precise measurement of the elongation of the bolt using an extensometer. The machine bolt used for the test is shown in Fig 1(a). Two circular plates of 20-mm thickness and 100-mm diameter, with three holes each, were used as a loading head in addition to three M-16 bolts. The test arrangement is shown in Fig 1(b). The results obtained from the tensile test of a standard M-20 bolt are show in Table 1.

**Table 1:** Bolt material properties.

Bolt	Young’s modulus (E) (kN/mm^2^)	Initial yielding stress (N/ mm^2^)	Ultimate stress (N/ mm^2^)
**M-20**	206.95	757	906

#### Preload on the bolts

**Fig 2:**
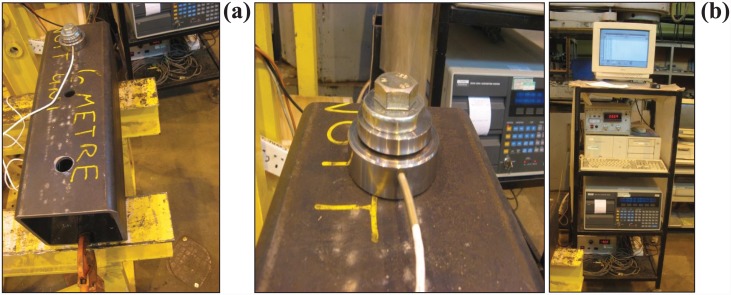
(a) Square hollow steel tube (200 X 200 mm) for preload test, (b) Load cell (left) and Data logger (right).

The degree of preload on a bolt has a significant effect on its overall performance. The preload induced in an M-20 EHB was recorded. The test set-up included a square hollow steel tube (200 × 200 mm) with a thickness of 10 mm, as shown in Fig 2(a).

A hole was then drilled into the steel tube to incorporate the EHB. The bolt was tightened at the nominal torque of 300 Nm using a torque wrench. The clamping thickness of the EHB was kept constant between the preload test and the pull-out test. A load cell was used to transmit the force into electrical signals and send data to a data logger. The load cell and the data logger used for preload testing are shown in Fig 2(b).

Mayer (1973) conducted an experiment to determine the preload on an A325 bolt. The author showed that most of the relaxation in the bolt occurs in the first five days of tightening, with the majority occurring in the first few minutes [10]. Based on these observations, the bolts used in this study were left to relax for five days after tightening. The data logger was set to record data every 10 seconds for the first five hours and every 30 minutes thereafter.

### Pull-out testing

#### The testing rig

**Fig 3:**
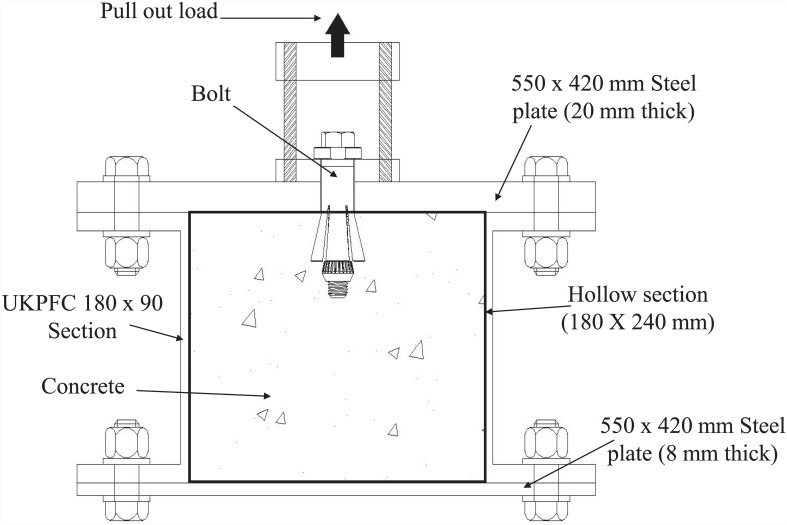
Cross section of testing rig.

The testing rig used for the pull-out test on the EHB and its respective components is shown in Fig 3. The testing rig comprised two steel plates—one on the top, and one on the bottom—with two steel channel sections in between on both sides, creating a hollow steel section inside. The dimensions and material properties of the testing rig elements were calculated based on the anticipated load for the pull-out test, to prevent face bending of the plates in the rig.

The dimensions of the plates and the side steel channel sections were designed to achieve a hollow section (180 mm deep and 240 mm wide). M-20 bolts were used to tighten the steel plates and channels together. A total of three rigs were used, one for each type of bolt to be tested (i.e., the EHB, the Hollo-Bolt and the standard bolt).

**Fig 4:**
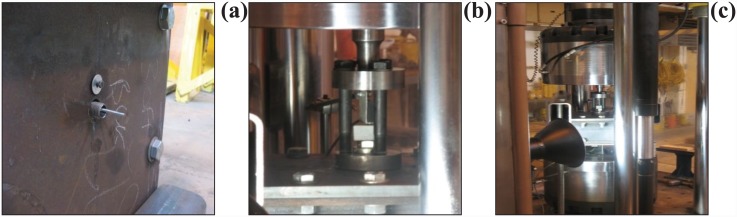
(a) M-3 rod encased in φ16 threaded hollow stud, (b) Loading head arrangement, (c) Pull-out test setup.

To measure the slip of the bolt, a φ16 threaded hollow stud was used in conjunction with an M-3 threaded rod, for which a φ18 hole was drilled in the bottom plate of the rig. After being fastened to the bolt, the M-3 rod was encased in the hollow stud, with both protruding through the bottom plate. Perfect contact between the hollow stud and the test bolt was achieved using nuts and washers at both ends of the stud, ensuring that no gap was left in between. The protruding M-3 rod encased in the φ16 threaded hollow stud at the bottom plate of the rig is shown in Fig 4(a). Square hollow steel tubes (100 × 100 mm, and 420 mm long) were used as supports for the rig. The height of these tubes was designed to allow enough room for the insertion of the bottom potentiometer, and a target for the video gauge camera.

As shown in Fig 4, a 4-mm threaded hole was drilled 30-mm away from the central hole in the bottom plate to attach an L-shaped aluminium plate; this was used as a support for the potentiometer to be attached at the bottom. Two circular steel plates (25-mm thickness and 100-mm diameter) were also used, along with three M-20 bolts (170-mm long), which were combined to act as a load head. One of the plates was used as a collar under the bolt head, while the other was attached to the loading mandrel of the pull-out testing machine. During the set-up of the pull-out test machine, three M-20 bolts were used to level the loading head for equal load distribution and to reduce the effect of any induced moment. This configuration of the bolts and two circular steel plates is shown in Fig 4(b).

Finally, two rectangular hollow steel tubes (150 × 100 × 1000 mm) were used as a reaction frame to hold the rig in place during pull-out testing. Four M-30 threaded steel rods with standard nuts were used at the corners of the rig to fix the two steel tubes to the ground. The testing rig and the instrumentation were designed to prevent face bending of the steel plates and channel sections. Consequently, the predominant failure mode was based on the test bolts themselves. Fig 4(c) shows the overall test set-up.

### The testing program

The bolts were tested in two sets (set A and set B) to ensure the reliability of the test data. Table 2 contains the details of the testing conducted in this study. The Hollo-Bolt and the EHB were tightened according to the nominal tightening torque, as indicated in the Lindapter Hollo-Bolt^®^ product brochure [11]. For the standard bolt, wooden planks and clamps were used to hold the bolt in place for concrete pouring.

**Table 2:** Testing program.

Bolt Type	Bolt Size	Bolt Grade	Bolt Length (mm)	Concrete Grade	Tightening Torque (N-m)
**Standard** Bolt	M-20	8.8	150	C40	-
**Hollo Bolt**	M-20	8.8	120	C40	300
**EHB**	M-20	8.8	150	C40	300

After tightening the bolts in the testing rig, and making the necessary arrangements for displacement measurements, the testing rig was cast with concrete according to standard practices. The specimen was then air-cured for seven days. After the concrete embedded within the bolt had set and gained the required strength, it was placed in the pull-out testing machine.

According to Macgregor (1997), concrete attains approximately 65–70% of the 28-day strength at the seventh day of curing [12]. Therefore, the specimens were tested at the seventh-day concrete strength, as concrete attains most of its strength in the first seven days. The failure mode for the rig was set as bolt fracture for all three types of bolts, by carefully selecting the dimensions of the rig elements, and calculating the effective load on the rig during pull-out tests. Additional specimens such as cubes, prisms and cylinders were cast alongside the specimens to determine the characteristics and strength of the concrete for the relative concrete mix used in this study.

Two 15-mm potentiometers were used to measure the top and bottom displacement of the entire rig. The top potentiometer was installed to measure total displacement of the specimen, while the bottom one was used to determine the slip of the bolt relative to the surrounding concrete. A small steel plate was attached to the M-3 rod protruding through the bottom plate hole. The steel plate was attached to maintain a levelled surface for the bottom potentiometer, which was used to record the slip of the bolt against the surrounding concrete. The potentiometers were connected to a data logger (Solartron/Schlumberger^®^ 3531D with Axis^®^ software) to record the displacement and slip values relative to load readings. A separate data logger (National Instrument SCXI-1000 with Servocon^®^ software) was also connected to the potentiometers for back-up recording of load displacement. Due to the placement of the top potentiometer, the latter data logger had to be removed after the ultimate failure point to avoid breakage. Thus, in addition to the potentiometers, a video gauge camera device was used to confirm displacement readings. This camera device allowed complete capture of the descending branch of the force-displacement curves.

The video gauge camera was initially levelled, and set at the middle of the testing rig, resting in the pull-out machine. It was focused to generate a clear picture of the rig on a data-recording laptop. As the camera recorded displacement in pixels, it was calibrated to obtain a conversion factor to convert the pixel readings into mm. A strong lamp was used to illuminate the targets on the testing rig to improve image clarity. The camera device was set up to provide readings of both total displacement (target-1) and slip (target-3) of the bolt, relative to the corresponding load.

### Concrete properties for set A and set B used in the pull-out testing

The concrete grade chosen for the pull-out tests was C40. For consistency between both sets of bolts, the same grade and concrete mix was used. The cement used was type II AL 32.5 R. Details of the mix design are given in Table 3.

**Table 3:** Concrete mix design.

Material	Kg/m^3^	Kg
Course Aggregate	1020	124.2
Fine Aggregate	735	89.5
Cement	440	53.6
Water	210	25.6

For each set (set A and set B), some cylinders, cubes and prisms were cast from the concrete batch to determine the concrete strength and other relevant properties. The details of the specimens cast, the tests performed and the results for the respective sets of bolts are given below.

For the first set of pull-out testing (set A), 16 standard cubes (100 × 100 mm) were cast, and cured in a water tank. Compressive strength was determined by standard compressive strength testing of the cubes on the 7th, 14th, 21st and 28th day of casting, to uncover trends in concrete strength. Four cubes were tested on each day of testing, and an average strength value was subsequently calculated. The compressive strength of the concrete with respect to concrete age is given in Table 4. The results from the water-cured cubes show that the concrete mix attained adequate strength.

**Table 4:** Compressive strength of water cured C40 concrete (set A).

Concrete age (Days)	Compressive strength (N/mm^2^)	Average saturated density (kg/m^3^)
7	38.4	2340
14	42.9	2340
21	44.5	2340
28	47.1	2340

For the second set of bolts (set B), twelve standard cubes (100 × 100 mm), three standard cylinders (φ150 mm, and 300 mm high) and three standard prisms (100 × 100 × 500 mm) were cast and all were air-cured, rather than water-cured. Compressive strength tests were conducted on the samples on the 7^th^, 14^th^, 21^st^ and 28^th^ day of casting. In addition to the compressive strength test, two more tests, namely PUNDIT (Portable Ultrasonic Non-Destructive Digital Indicating Tester) and ERUDITE (electronic resonant frequency test system), were also conducted on the prisms to measure the dynamic modulus of the concrete.

According to EC2, the dynamic modulus of elasticity of concrete can be found using the following equations for PUNDIT and ERUDITE tests:

For PUNDIT:

$$E_{D}=\;\frac{V^{2}[\rho (1+\mu )(1-2\mu )]}{\left(1-\mu \right)}$$ (1)

where *E*_*D*_ = dynamic modulus of elasticity (kN/mm^2^), ρ = density of the prism (kg/m^3^), *μ* = Poisson’s ratio (taken as 0.24) and *V* = pulse velocity (m/s).

For pulse velocity:

$$V=\;\frac{L}{\Delta t}$$ (2)

where *L* = length of the specimen (m) and Δ*t* = time taken by the pulse (μsec).

For Erudite:

$$E_{D}=\;4\times l^{2}\times F_{L}{}^{2}\times \rho$$ (3)

where *E*_*D*_ = dynamic modulus of elasticity (kN/mm^2^), *l* = length of the prism (m), *F*_*L*_ = resonance frequency (Hz) and *ρ* = density of the prism (kg/m^3^).

For the static modulus of elasticity of concrete (*E*_*cm*_):

$$E_{cm}=\;\frac{E_{D}}{1.05}$$ (4)

where *E*_*cm*_ = static modulus of elasticity of concrete (kN/mm^2^) and *E*_*D*_ = dynamic modulus of elasticity (kN/mm^2^).

The concrete properties for the concrete used in set B are given in Table 5.

**Table 5:** Concrete properties for the concrete mix (Set B).

Concrete age (Days)	Compressive strength (N/mm^2^)	Dynamic modulus of elasticity (E_D_) (kN/mm^2^)		Static modulus of elasticity (E_cm)_ (kN/mm^2^)	
		*PUNDIT*	*Erudite*	*PUNDIT*	*Erudite*
7	35.5	36.2	34.4	35.7	34.0
14	40.0	37.0	35.2	37.1	35.3
21	41.0	-	-	-	-
28	41.5	37.4	35.6	37.7	36.0

The average dynamic modulus (*E*_*D*_) value was 35.96 kN/mm^2^, while that of the static modulus (*E*_*cm*_) was 36 kN/mm^2^.

### Force-displacement analysis

The results obtained from the pull-out tests on the EHB and its components (i.e., the Hollo-Bolt and standard bolt shank with anchor head) were then analysed, and force-displacement graphs were generated. As mentioned previously, the top potentiometer was used to measure the total displacement of the bolts in the concrete specimen, while the bottom potentiometer was used to measure the slip of the bolt against the concrete specimen. As the failure mode was set as bolt fracture, the total displacement of the whole specimen consisted of two parts: the slip of the bolt and the bolt elongation. The bolt elongation was calculated by simply subtracting the slip of the bolt from its total displacement, as shown in Eq (6):

Total displacement (δt)=Slip(δs)+Bolt elongation(δb) (5)

For Bolt Elongation:

Bolt elongation(δb)=Total displacement (δt)−Slip(δs) (6)

Force-displacement graphs for the three types of displacement (total displacement, slip and bolt elongation) were then plotted for the EHB and its components. These graphs were generated to ascertain the bonding and anchorage characteristics of the bolts under consideration. These graphs were also used to determine the ultimate failure point of the bolts.

## Test Results and Discussions

This section discusses the results of the pull-out tests described earlier. The force-displacement curves for the two sets of bolt testing are presented first. The trends in stiffness for the EHB are subsequently examined. Finally, the force-displacement curves of the M-20 bolts are compared to those of the M-16 bolts, characterised previously by Pitrakkos [9], to evaluate bolt behaviour relative to changes in diameter. All other parameters were kept identical; only the diameter of the bolt was changed.

### Preload test results

**Fig 5:**
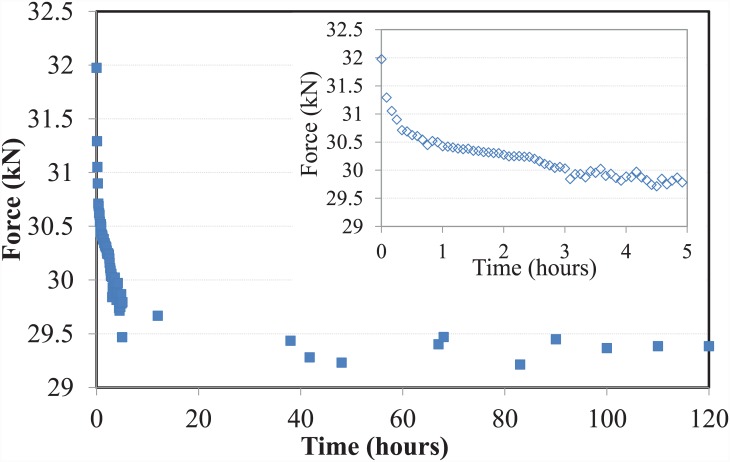
Preload test result (first 5 hours (inset) and days).

After leaving the test set-up for five days, the readings from the data logger were collected and analysed. As anticipated, most of the relaxation occurred within the first few hours, after which the bolt preload stabilised. The filtered results of the preload test, and the relaxation of the bolt over the first five hours and the first five days are shown in Fig 5. Close scrutiny of the test results shows that most of the relaxation occurred within the first few hours.

**Fig 6:**
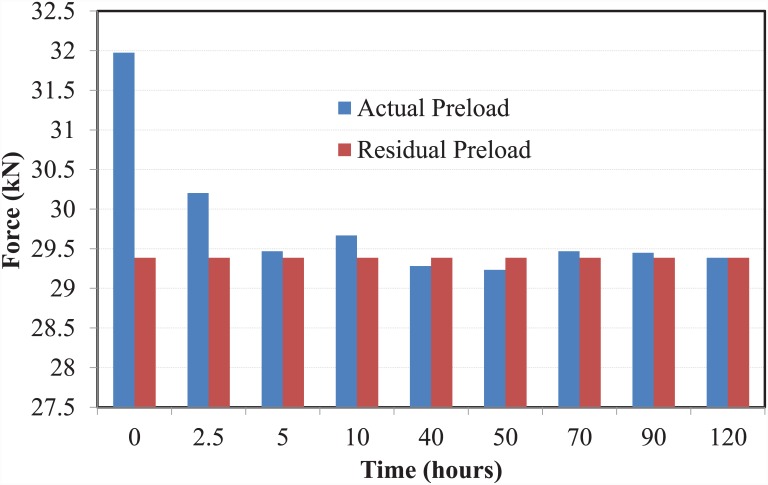
Comparison of actual and residual preload.

The preload induced on the bolt after five days (120 hours) of tightening was found to be 29.4 kN. A bar chart comparing the residual preload against the actual preload is shown in Fig 6. The test revealed that approximately 92% of the relaxation was achieved within the first two hours of tightening of the bolt.

### Pull-out test results

As described previously, the EHB and its components were tested in two sets to ensure the reliability of results. Data obtained from both the data loggers and the video gauge camera device were analysed and compared. The force-displacement graphs for both the M-20 EHB as a whole and its individual components are presented below.

### The bond and anchorage mechanism of EHB

**Fig 7:**
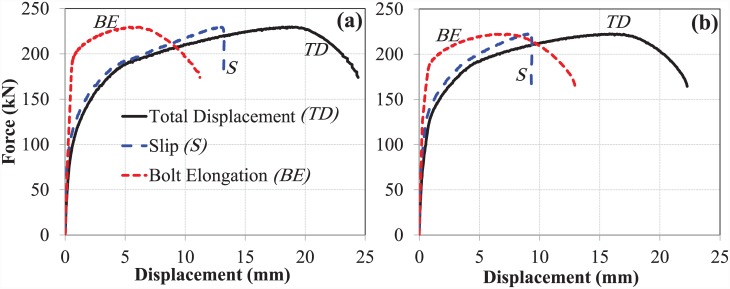
Force-displacement curves for bond and anchorage component. (a) set A, and (b) set B.

The bond and anchorage component (comprising a standard bolt shank with an anchor head attached to the bottom) of the EHB assembly were tested against pull-out load, and the results recorded by both potentiometers were plotted for each of the sets (set A & B). The force-displacement curves for total displacement, bolt slip and bolt elongation of the bond and anchorage components of both sets are shown in (Fig 7a & 7b).

Both test sets followed a similar trend in terms of displacement values, and were found to be in good agreement with one another. As fracture of the bolt shank was used as the mode of failure for both sets, it is notable that after failure, no slip displacement of the bolt against the concrete was observed, although elongation and yield still occurred.

#### The expanding sleeves component (hollo-bolt)

**Fig 8:**
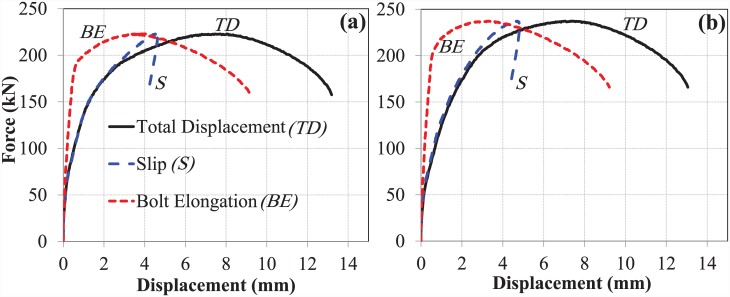
Force-displacement curves for hollo-bolt HB-20. (a) set A, and (b) set B.

The Hollo-Bolt, as the expanding sleeves component of the EHB assembly, was tested against pull-out load, and the results obtained from the potentiometers were plotted for both sets of bolts. The force-displacement curves for total displacement, bolt slip and bolt elongation of the Hollo-Bolt (sets A and B) are shown in (Fig 8a & 8b).

The force-displacement trends obtained for the two M-20 Hollo-Bolts showed good agreement. Like the bond and anchorage component, the Hollo-Bolt prevented slip against the concrete after the ultimate load point, although the bolt continued to yield, leading to bolt failure. Although the slip of the bolt began to recede after the ultimate load point, indicating minor settling of the bolt, closer inspection of the initial displacements suggests that the bolt and its slip relative to surrounding concrete had almost identical stiffness. The total displacement and the slip of the Hollo-Bolt were smaller than that of the bond and anchorage component: total displacement was approximately 40% lower, and the slip approximately 60% lower. The extra length of the bolt shank enhances the bolt’s performance against tensile load and makes it more resistant to the slippage.

#### The overall component (EHB)

**Fig 9:**
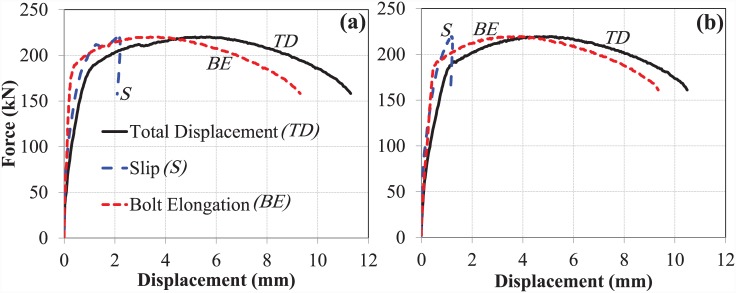
Force-displacement curves for EHB-20. (a) set A, and (b) set B.

Two EHB were tested in the pull-out testing machine in a manner similar to the bond and anchorage component and the expanding sleeves component. Measurements of displacement were then plotted against load data. The force-displacement curves for total displacement, bolt slip and bolt elongation of the EHB as a whole (sets A and B) are shown in (Fig 9a & 9b).

The curves for both sets of bolts showed good agreement. The degree of total displacement and bolt elongation was roughly equivalent to that of the Hollo-Bolt, although the slip of the bolt in this case was reduced by almost 50%. This is attributable to the additional anchorage and bond provided by the anchor head and the EHB. Thus, it is clear that the increased length of the bolt shank and the provision of an anchor head in the EHB enhance the bolt’s performance against tensile load, and render it more resistant to slippage than the Hollo-Bolt by facilitating a stronger bond with the surrounding concrete.

### Failure mode of the bolts

**Fig 10:**
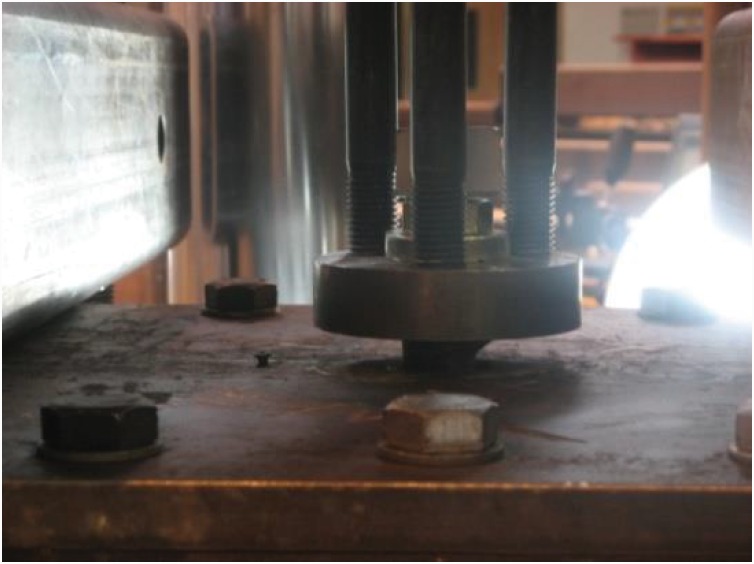
Displacement of EHB.

Following pull-out testing, the concrete specimens were removed from the testing rig and visually analysed for any cracks or breakage. The length of the fractured bolt was measured from the bottom of the bolt head to the necking point. This length represents the distance between the weakest point of the bolt and the bolt head. Fig 10 shows the top displacement of the EHB at a point during the pull-out test.

The details of the failure mode of both test sets are given below.

#### Failure mode of set A

**Fig 11:**
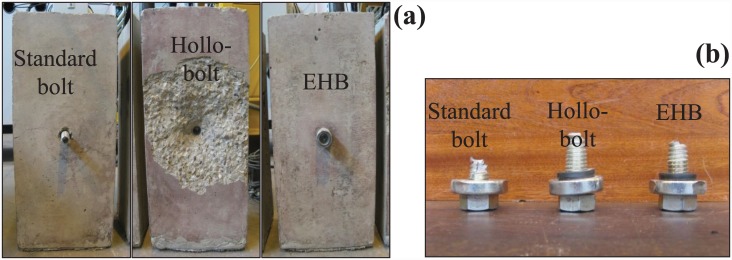
(a) Failed concrete specimens, and (b) Bolt shanks after failure (set A).

The concrete specimen of the bond and anchorage component exhibited a transverse splitting crack around the bolt, reaching all the way across the specimen face. In the case of the Hollo-Bolt, failure was caused by breakage of a concrete cone. The cone developed as a result of the angle created between the edge of expanding sleeves and the surface of the concrete. No significant cracks were observed on the EHB specimen. This was attributable to the efficient bond and anchorage of the EHB to the surrounding concrete. The failed concrete specimens from set A are shown in Fig 11(a). The bolt shanks of the standard bolt, Hollo-Bolt and EHB after failure are shown in Fig 11(b).

#### Failure mode of set B

**Fig 12:**
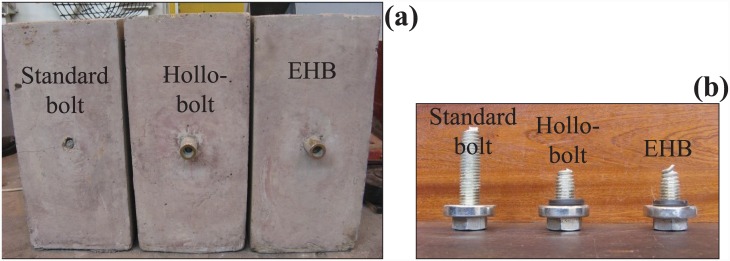
(a) Failed concrete specimens, and (b) Bolt shanks after failure (set B).

In set B, a similar transverse crack pattern was observed on the bond and anchorage component specimen. The failed Hollo-Bolt specimen exhibited substantial radial cracking around the bolt, although the concrete around the bolt did not break open entirely. The EHB specimen in set B did not show any significant cracking. The failed concrete specimens and the failed bolt shanks are shown (Fig 12a & 12b), respectively.

**Fig 13:**
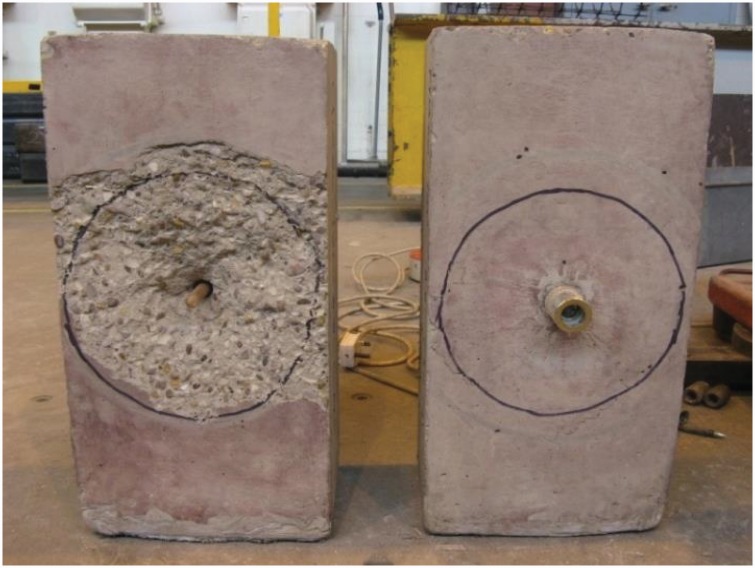
Crack diameter for hollo-bolt specimens; set A (left) & set B (right).

The diameter of the crack formed around the Hollo-Bolt was marked and compared to the crack diameter of the specimen in set A. The diameter of the crack was 214 mm. Fig 13 shows the size of the marked crack diameter for specimens in sets A and B.

The length of the bolt shank after bolt failure for the three specimens in both sets is shown in Table 6.

**Table 6:** Length of failed bolt shanks.

	Bond and anchorage component	Expanding sleeves component (Hollo-bolt)	Overall component (EHB)
**Set A**	15 mm	39 mm	36 mm
**Set B**	78 mm	39 mm	41 mm

### Comparison of M-20 bolts with M-16 bolts

As mentioned previously, data on the performance of M-16 bolts in pull-out testing were obtained from a previously published report [9]. Concrete grade C-40 was also used for the M-16 bolts. As mentioned earlier, all other parameters were also identical; only the diameter of the bolt was changed. The slip and elongation at both bolt sizes were then compared for the EHB and its components to investigate the behaviour of the bolts with respect to the change in diameter.

#### The bond and anchorage component

**Fig 14:**
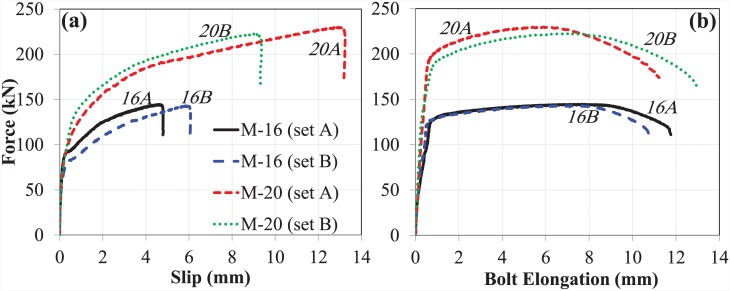
(a) Slip, and (b) bolt elongation comparison of M-16 and M-20 (bond and anchorage component).

The length of the bolt shank for both bolt sizes was kept constant. However, due to the increased diameter of the bolt shank and the increased size of the anchor head, the load-carrying capacity and the relative slip against concrete of the M-20 bolt was enhanced. (Fig 14a & 14b) show comparative analysis of the slip and bolt elongation, respectively, of the bond and anchorage component for both bolt sizes.

The curves show that the bond and anchorage component of the M-20 bolt has much greater stiffness than that of the M-16 bolt. The load-carrying capacity of the M-20 bolt was approximately 50% greater than that of the M-16 bolt. However, no significant difference in bolt elongation, or ductility, was observed between the two bolt sizes. The slip of the M-20 bolt, however, was almost double that of the M-16 bolt.

#### The expanding sleeves component (hollo-bolt)

**Fig 15:**
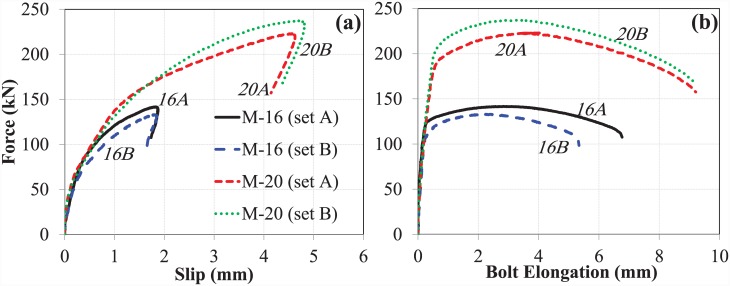
(a) Slip, and (b) bolt elongation comparison of M-16 and M-20 (hollo-bolt).

Force-displacement curves for slip and bolt elongation were similarly generated for the expanding sleeves (or Hollo-Bolt) components of the M-20 and M-16 bolts. A comparison of slip and bolt elongation between the two bolt sizes is shown in (Fig 15a & 15b), respectively.

The pattern of displacement was similar for both Hollo-Bolt sizes, as shown in Fig 15. In this case, the load-carrying capacity of the M-20 bolts was increased by up to 60%. The stiffness and ductility of the bolt increased with size.

#### The overall component (EHB)

**Fig 16:**
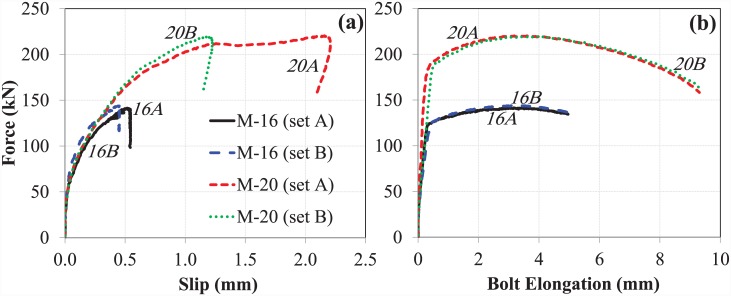
(a) Slip, and (b) bolt elongation comparison of M-16 and M-20 (EHB).

In addition to the comparison of individual EHB components, a comparison of the entire EHB at both sizes was made. Comparative force-displacement graphs were generated for slip and bolt elongation, as shown in (Fig 16a & 16b), respectively.

Overall trends in slip and bolt elongation were similar for both sizes of EHB. The M-20 EHB was found to be stiffer than the M-16, as expected. The ultimate load carried by the M-20 bolts was 54% greater than that carried by the M-16 bolts. It is also evident from the non-linear curves in Fig 16 that the ductility of the bolt increases with increasing diameter.

Analysis of the M-20 EHB revealed that the anchor head at the end of the bolt shank, and the increased length of the bolt, served to enhance resistance against slippage relative to the Hollo-Bolt. Furthermore, the bond of the bolt with the surrounding concrete was found to be much stronger than the tensile strength of the bolt shank. A comparison of the M-20 EHB and M-16 EHB shows that bolt stiffness increases with increasing diameter. The load-carrying capacity of the M-20 EHB was found to be approximately 54% greater than that of the M-16 EHB.

## Proposed Spring Model for the EHB

In this study, a spring model was used to compare the stiffness of the EHB with that of its components. This model can be used to understand the individual behaviour of each component under load, and to elucidate the relative contribution of each component to the pattern of the load-carrying mechanism.

**Fig 17:**
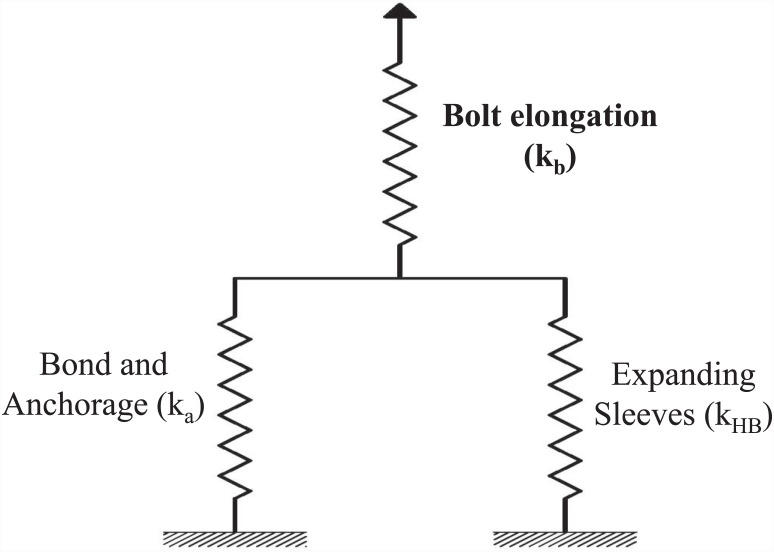
Proposed spring model for EHB.

A spring model was proposed by Pitrakkos and Tizani [13] for comparison of stiffness of the EHB with its individual load-carrying components. In this model, the stiffness of various components of the EHB are combined based on the spring theory, and compared with the stiffness of the EHB itself. The configuration of the proposed spring model is shown in Fig 17.

The three components depicted in Fig 17, when combined appropriately, should produce an overall effect identical to the EHB itself. In the case of the Hollo-Bolt, the bolt length is too short to achieve any significant bonding with the surrounding concrete, and load is predominantly carried by the expanding sleeves. Thus, the Hollo-Bolt itself can be considered as the expanding sleeves component of the EHB. In the case of the standard bolt shank, where the anchor head is attached at the bottom, the load is predominantly carried by the bond between the bolt and the concrete, and the anchorage is provided by the anchor head. Consequently, the bolt shank and anchor head mechanism can be considered as the bond and anchorage component of the EHB.

According to the spring theory, the stiffness of the individual components presented in the spring model can be combined to give an equivalent stiffness, as shown below:

Parallel spring:

$$k_{e1}=\;k_{a}+k_{HB}$$ (7)

Series spring:

1ke= 1ke1+1kb (8)

where k_a_ = stiffness of the bond and anchorage component, k_HB_ = stiffness of the expanding sleeves component, k_b_ = stiffness of the bolt elongation component, k_e1_ = combined stiffness of the springs in parallel and k_e_ = equivalent stiffness of the spring mechanism.

For the bolt elongation component, two options were available. One solution was to obtain the bolt elongation experimentally by subtracting the slip of the EHB from its total displacement, as described in Eq (5). An alternative solution was to apply the bolt model presented by Swanson [14]. According to Swanson, the bolt elongation can be obtained using the stiffness model, as shown in Table 7.

$$K_{b}=\;\frac{EA}{L}$$ (9)

where K = initial stiffness of the bolt (kN/mm), E = modulus of elasticity of the bolt (kN/mm^2^), A = cross-sectional area of the bolt (mm^2^) and L = gripping length of the bolt (mm).

**Table 7:** Bolt stiffness model (Swanson, 2001).

Bolt Force (B)	Bolt Stiffness (K)
0≤ B <B_*o*_	K_*b*,*1*_ = 1.00 K_*b*_
B_*o*_ ≤ B < 0.85 B_*n*_	K_*b*,*2*_ = K_*b*_
0.85 B_*n*_ < B < 0.9 B_*n*_	K_*b*,*3*_ = 0.10 K_*b*_
0.9 B_*n*_ < B < B_*fract*_	K_*b*,*4*_ = 0.02 K_*b*_

**Fig 18:**
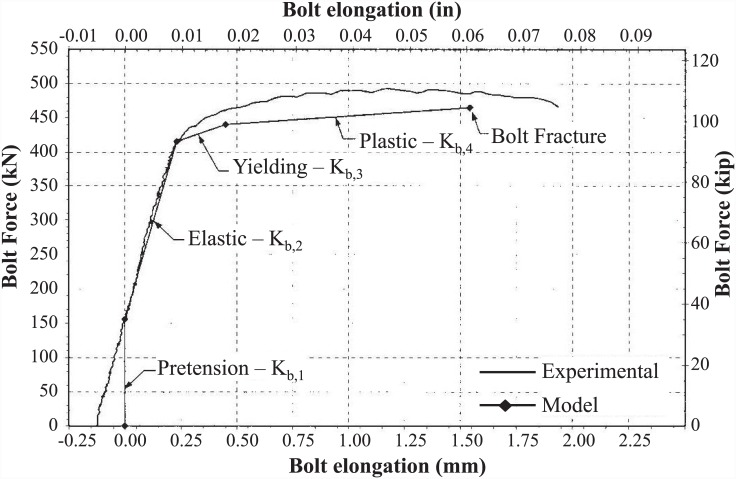
Bolt elongation model (Swanson, 2001).

Swanson (2001) verified this model by performing T-stub component tests and individual bolt tests [14]. Values obtained using the model showed good agreement with his experimental values, as shown in Fig 18.

Both methods were used for analysis of bolt elongation and were verified in relation to the stiffness of the EHB.

### Tri-linear idealisation

**Fig 19:**
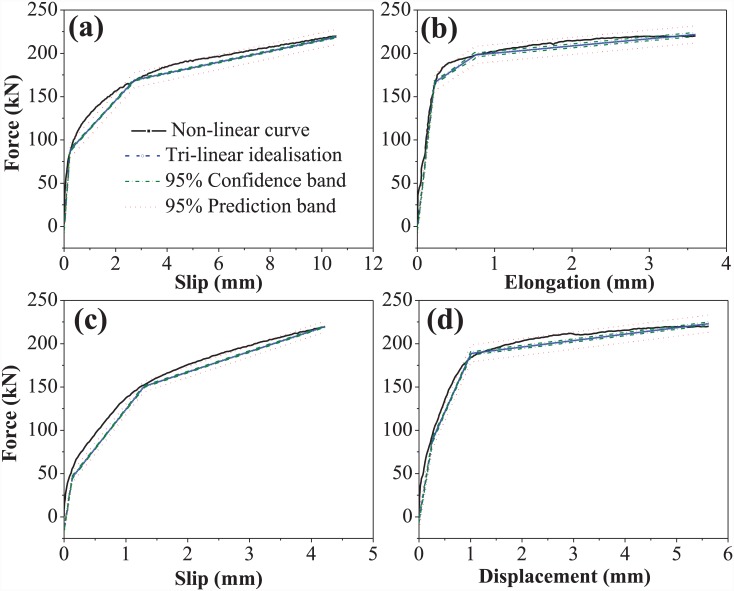
Tri-linear idealisation of (a) bond and anchorage component for M-20 bolt slip, (b) expanding sleeves component of HB-20 slip, (c) bolt elongation of EHB-20, and (d) total displacement of EHB-20.

The non-linear force-displacement curves generated from pull-out testing were idealised in a tri-linear manner for piecewise analysis of the spring model through dynamic linear regression analysis: Hooke’s law and spring theory can only be applied to linear data sets. Graph-plotting software was used to idealise the non-linear curves for set A. The plotted data were validated against the conventional 95% confidence intervals and the classical 95% prediction bands, to ensure that the tri-linear curves obtained were within the defined limits, and to determine the precision of the idealisation. Fig 19(a) shows the tri-linear idealisation of the slip curve for the bond and anchorage component of the M-20 bolt.

The non-linear slip curves for the Hollo-Bolt and bond and anchorage component of the EHB, and the bolt elongation curves of the EHB, were also idealised as tri-linear curves. Values obtained from the application of the bolt elongation model presented by Swanson [14] were compared with experimental values. Stiffness was calculated from each curve by breaking the curves down into force ranges. Fig 19(b) shows the tri-linear idealisation of the expanding sleeves component of HB-20 slip for set A.

The resulting curve obtained from the addition of the component’s tri-linear curves was subsequently compared to the tri-linear curve of total EHB displacement for validation of the proposed spring model. Tri-linear idealisation curves for EHB-20 bolt elongation and total displacement are shown in (Fig 19c & 19d), respectively.

The tri-linear curves generated were subsequently summated. Force ranges were selected and stiffness was calculated for each segment of each curve. Stiffness values for each component were summated using the conventional rule of addition of stiffness in parallel and in series. Two different models were generated: one for each of the bolt elongation models.

### Swanson’s bolt elongation model

The bolt elongation model proposed by Swanson [14] was implemented for the bolt elongation mechanism, and a tri-linear curve was obtained representing the combined assembly of the individual components of the EHB.

#### Tri-linear curves for the components of the EHB

**Fig 20:**
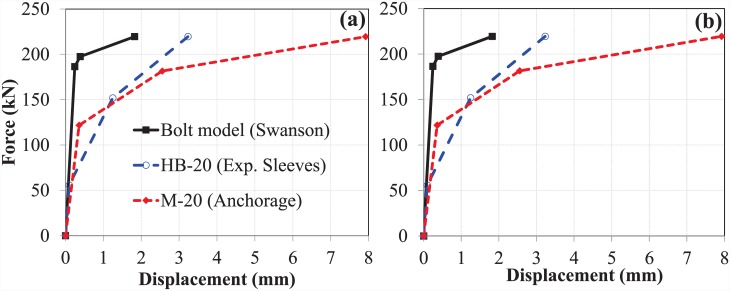
Components of EHB with Swanson’s bolt elongation model. (a) set A, (b) set B.

The tri-linear curves obtained for the three components of the EHB, where the bolt elongation component was obtained from Swanson’s bolt elongation model, are shown in Fig 20.

#### Assembly of the components

**Fig 21:**
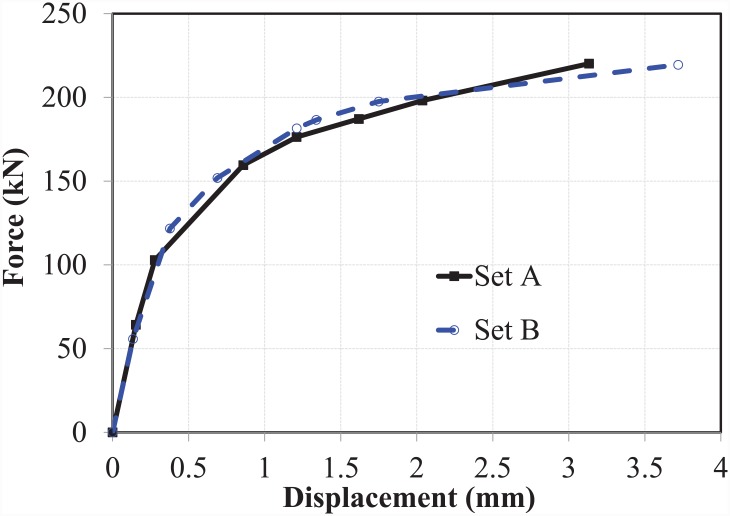
Components of EHB combined through spring theory (Swanson’s bolt model).

Whereas the expanding sleeves and bond and anchorage components were in parallel, the bolt elongation component was in series. These components were combined, and the resulting multi-linear curve was compared to the tri-linear force-displacement curve of the EHB. The assembly of the components for both sets, where the bolt elongation was derived from Swanson’s model, is shown in Fig 21.

#### Comparison of the EHB with its components

**Fig 22:**
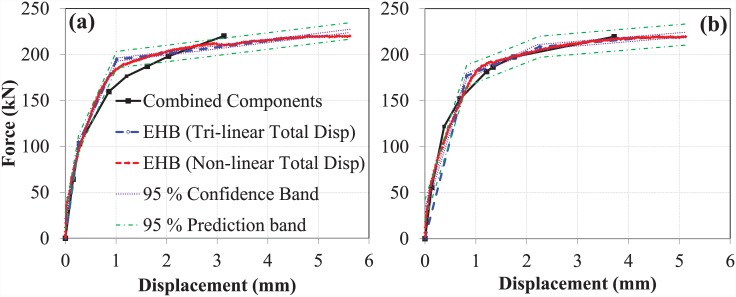
Comparison of EHB with its components (Swanson’s bolt model). (a) set A, (b) set B.

The multi-linear curve obtained through the addition of the EHB components was then compared to the tri-linear and non-linear total displacement curves for the EHB, as shown in Fig 22.

The model obtained for the combined components was found to obey the 95% confidence bands and the 95% prediction intervals, with a slight deviation in the semi-plastic phase. The initial stiffness of the combined components was found to be roughly equivalent to that of the EHB. Shortly before reaching the yielding point of the bolt, the combined component model was seen to deviate minutely from the EHB curve. The difference between the ultimate failure points can be explained by the fact that the EHB curve was generated based on experimental data: this incorporates both tensile force and minor bending, while only tensile force is accounted for in the bolt elongation model. Furthermore, once the bolt reaches the ultimate failure point during pull-out testing, the load begins to decrease and neck until the bolt fracture point. This may also contribute to the observed differences in ductility in both tri-linear curves.

### Experimental bolt elongation model

Swanson’s bolt elongation model was replaced with the EHB bolt elongation data recorded from pull-out testing. The three components of the EHB were re-analysed and combined according to spring theory.

#### Tri-linear components of the EHB

**Fig 23:**
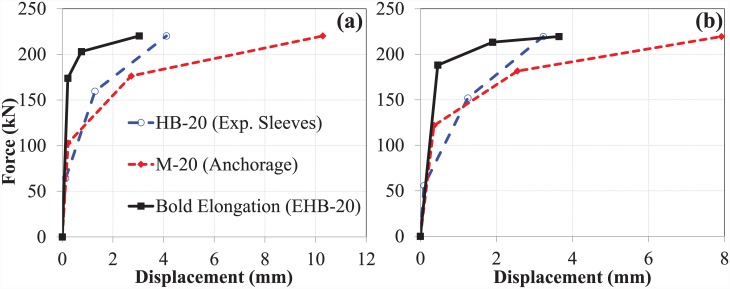
Components of EHB with experimental bolt elongation model. (a) set A, (b) set B.

Using the bolt elongation curve obtained from experimental results, the bolt elongation data for the bond and anchorage, and expanding sleeves components were plotted and combined based on spring theory. The tri-linear curves for the individual components, obtained after tri-linear regression analysis, are shown in Fig 23.

#### Assembly of the components

**Fig 24:**
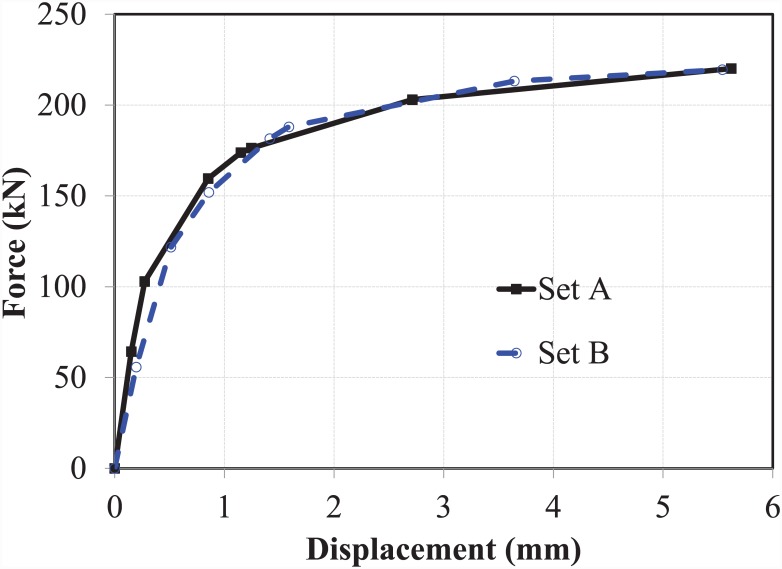
Components of EHB combined via spring theory (Experimental bolt model).

After combining the tri-linear curves obtained from tri-linear regression analysis, using the bolt elongation mechanism obtained from the experimental results of EHB pull-out testing, a multi-linear curve was generated. The curves representing the combined assembly of the components for both sets are shown in Fig 24.

#### Comparison of the EHB and its components

**Fig 25:**
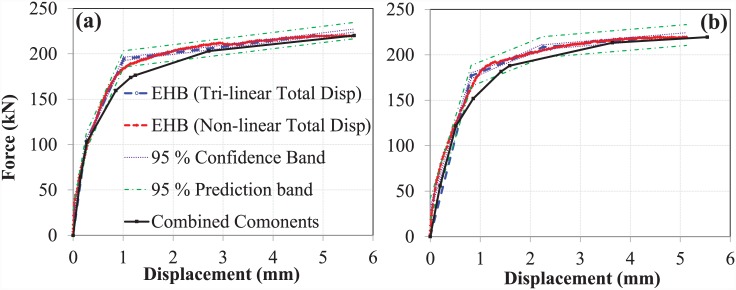
Comparison of EHB with its components (Experimental bolt model). (a) set A, (b) set B.

The multi-linear curve for combined components was then compared to the experimental non-linear and tri-linear force-displacement curve of the EHB, as shown in Fig 25. The combined component models were largely within the 95% confidence bands and 95% prediction intervals, with the exception of a small portion in the semi-plastic phase. As in the previous model (Swanson’s bolt elongation curve), the combined initial stiffness (during the linear elastic phase) of the EHB components was identical to that of the EHB itself. Contrary to the previous model, the stiffness of the EHB during the plastic phase was also almost identical to that of the combined components. Data obtained from the component model were largely comparable to those obtained from the non-linear experimental curve of the EHB. Furthermore, the differences in ductility values derived from the component model and the actual EHB test during the plastic phase were minimal in the case of the experimental bolt elongation model.

Analysis using the proposed spring model revealed that the stiffness of the EHB is comparable to that of its combined components. Furthermore, the stiffness model based on experimental EHB elongation data, showed greater compliance with the spring model than with the bolt elongation model presented by Swanson [14]. A potential explanation is that the latter was originally prepared for the M-16 bolt size, and hence minor disagreement could not be considered negligible.

## Conclusions

This component-based study included the testing of M-20 bolts (EHB and its components), individual and collective analysis, and parametric comparison with M-16 bolts. Various non-linear force-displacement curves (total displacement, slip and bolt elongation) were generated, and a spring model to predict the stiffness of the extended Hollo-Bolt was proposed. The proposed model was validated by comparing the stiffness of combined component model to that of the extended Hollo-Bolt. Based on the results of current study, the following conclusions have been made:

1. The initial relaxation of the EHB occurs within the first four to five days of tightening of the bolt. The residual preload prevailed after that time period. The majority of the relaxation occurred within the first three hours of tightening.
2. The force-displacement curves obtained for the EHB and its components in both sets exhibited good agreement, demonstrating reliability of the test results. The curves generated provide insight into the behaviour of the EHB and its components, specifically the total displacement of the bolt, the slip of the bolt against the concrete specimen, and the elongation of the bolt shank.
3. Comparison of the M-20 and M-16 bolts revealed an increase of approximately 50% in the load-carrying capacity of the M-20 bolts. These results demonstrate that stiffness, ductility and load-carrying capacity of the EHB and its components increase with increasing bolt diameter.
4. The failure mode of the bolts was identical against the three types of displacements mentioned, however. The failure surface area of the Hollo-Bolt specimen was found to increase with increasing bolt diameter.
5. The spring model used to predict the stiffness of the EHB according to its individual mechanisms was validated through summation of the stiffness of all three components, and through comparison of the final multi-linear graph with the tri-linear and non-linear force-displacement curves obtained from the EHB.
6. The two methods used to characterise the bolt elongation mechanism generated highly similar results, although the experimental bolt elongation mechanism obtained from the EHB test results was found to be more appropriate for the model. The good agreement between the stiffness of the EHB, and the combined stiffness of its components, validates the use of this model for predicting the behaviour of the EHB based on its individual mechanisms.

To facilitates investigation of the load transfer mechanism of the blind bolt, a component-based finite element model be generated in future works for the individual components of the EHB. Further experimental as well as parametric studies may also be performed for various other parameters, such as bolt shank length, bolt grade, concrete grade, expanding sleeves opening, gripping length of the bolt shank, and anchor head shape and size can be varied to assess their respective effects on EHB behavior. Furthermore, the EHB should also be tested against cyclic loading, and parametric changes should be taken into consideration to facilitate the evaluation of a blind bolt’s response to earthquake loading.

## Abbreviations

SHS: Structural Hollow Section
CFST: Concrete-Filled Steel Tubular Columns
EHB: Extended Hollo-Bolt
RMH: Reverse Mechanism Hollo-Bolt
E: Young’s Modulus of Elasticity
UKPFC: Parallel Flange Channels
δt: Total Displacement
δs: Slip
δb: Bolt Elongation
E_D_: Dynamic Modulus of Elasticity of Concrete
E_cm_: Static Modulus of Elasticity of Concrete
ρ: Density of prism
μ: Poisson’s ratio
V: Pulse velocity
F_L_: Resonance frequency
k_a_: Stiffness of the Bond and Anchorage Component
k_HB_: Stiffness of the Expanding Sleeves Component
k_b_: Stiffness of the Bolt Elongation Component
k_e1_: Combined Stiffness of the Springs in Parallel
k_e_: Equivalent Stiffness of the Spring Mechanism
K: Bolt Stiffness
B: Bolt Force
A: Cross-sectional Area of the Bolt
L: Gripping Length of the Bolt
